# Dr. George Strasenburgh

**Published:** 1912-02

**Authors:** 


### OBITUARY

Our readers are requested to send notices of deaths of physicians in western New York, or who have resided elsewhere but have been graduates of schools of this region or who have otherwise been identified with this region. State residence, date of death, age, cause of death, school of graduation, and other items of interest.

DR. GEORGE STRASENBURGH died at his home in Morton, N. Y., December 26, 1911, aged 79, of apoplexy. He was a graduate in arts of Queen's College, of Kingston; in theology of Toronto University, and in medicine of the University of Buffalo, 1882. While the greater part of his life was spent in the Presbyterian ministry, he had practiced medicine in Morton for the last ten years and was health officer of the town. He is survived by his wife and by three sons, Dr. Frederick A. Strasenburgh of Avon, Robert J. Strasenburgh, a pharmacist of Rochester, and Sidney G. Strasenburgh of Morton.

